# High-Grade Renal Trauma Due to Blunt Injury: Do All Require Surgical Intervention?

**DOI:** 10.3390/diagnostics16203313

**Published:** 2026-10-12

**Authors:** Anda Hogea, Ioan Scarneciu, Marius Moga, Mircea D. Hogea, Florin Lucian Sabou, Adriana Prahoveanu, Simona Grigorescu, Andrada Nicolau

**Affiliations:** Faculty of Medicine, Transilvania University of Brasov, 500036 Brasov, Romania; anda.szasz@unitbv.ro (A.H.);

**Keywords:** high-grade renal trauma, blunt injury, management, outcomes, retrospective study, grade III, grade IV, grade V

## Abstract

**Background:** Renal trauma management has increasingly shifted toward non-operative treatment, particularly in hemodynamically stable patients. Advances in imaging, resuscitation, and monitoring have improved the outcomes of conservative management, even in high-grade injuries. Blunt trauma, most commonly from falls, is the leading cause, and renal injuries often occur within the context of polytrauma involving multiple organ systems. Management is primarily guided by hemodynamic stability, overall clinical condition, existing comorbidities, and the severity of injury as determined by the American Association for the Surgery of Trauma (AAST) grading system. This study aimed to describe the management strategies and clinical outcomes of patients with high-grade blunt renal trauma (AAST grades III–V), with particular emphasis on the use of conservative management in selected patients. **Methods:** This study is a retrospective analysis conducted in our department (Emergency Clinical County Hospital in Brasov, Romania) to identify the data of the patients admitted with high-grade blunt renal trauma from 1 January 2019 to 31 December 2025. **Results:** A total of 58 patients with blunt renal trauma (grade III or higher) were identified. According to the AAST classification, the most common injuries were grade III (35 patients), followed by grade IV (11 patients) and grade V (12 patients). The majority of cases (68.97%) were treated conservatively, and the overall mortality rate was 8.62%. **Conclusions:** This study highlights the importance of individualized management in renal trauma, supporting conservative treatment in hemodynamically stable patients. Hemodynamic stability, renal injury grade, and the extent of associated injuries should guide treatment decisions. Further research is needed to refine treatment protocols and assess long-term outcomes.

## 1. Introduction

Renal trauma accounts for approximately 1–5% of all traumatic injuries and up to 10% of abdominal trauma cases, making the kidney the most frequently injured organ of the genitourinary system. The majority of cases occur in young male patients and result from blunt mechanisms (approximately 80–90%), most commonly falls, road traffic accidents and violence [1,2].

Blunt abdominal trauma most commonly involves solid organs such as the liver and spleen. Owing to their anatomical location and relative mobility, the kidneys are particularly susceptible to injury during high-energy traumatic events [3].

Renal injuries are classified according to the AAST grading scale, which categorizes lesions from grades I to V based on the extent of parenchymal damage, involvement of the collecting system, and vascular injury. Low-grade injuries (I–II) account for approximately 70–85% of cases and are managed almost exclusively with non-operative strategies. Over the past two decades, non-operative management has become the standard of care for hemodynamically stable or stabilized patients, including a substantial proportion of high-grade (grade III-IV) injuries, with reported success rates exceeding 80–90% in specialized centers. This standardized grading system plays a very important role in guiding clinical decision-making and determining the most appropriate management strategy [4,5].

Renal injuries contribute significantly to patient morbidity and, in the presence of associated multisystem trauma, may also increase mortality. A thorough understanding of the mechanisms of injury, classification systems, and evolving management strategies for blunt renal trauma is essential for trauma surgeons, urologists, and emergency physicians to optimize outcomes and minimize long-term renal complications [6].

The majority of patients present with classic signs such as flank pain and gross hematuria, others may have nonspecific or no symptoms, particularly in the context of complex polytrauma. The absence of hematuria does not exclude significant renal injury, making imaging crucial for accurate diagnosis and injury grading. Contrast-enhanced computed tomography remains the gold standard imaging method, allowing evaluation of renal parenchymal damage, urinary extravasation, active bleeding, and associated injuries [7,8].

Management is primarily guided by hemodynamic stability, overall clinical condition, existing comorbidities, and the severity of injury, as determined by the American association for the Surgery of Trauma (AAST) grading system [8]. The optimal management of renal trauma, particularly high-grade injuries, has been a subject of debate for a long time. Nonoperative management is now the standard of care for solid visceral injuries of the liver and spleen, with well-documented success. Consequently, there has been a growing shift toward conservative management of renal injuries, mirroring the favorable outcomes [9].

While low-grade renal injuries are well recognized to respond favorably to observation alone, the applicability of this conservative approach to high-grade renal injuries—often occurring in critically ill patients—remains uncertain. Multiple studies have shown that, in carefully selected patients, non-operative management is safe and effective for grade IV injuries, offering high rates of renal preservation and low rates of delayed intervention [1,10].

The aim of this study was to evaluate the clinical outcomes and management strategies of patients with high-grade renal trauma following blunt abdominal injury, with particular emphasis on the use of conservative management in selected patients.

This methodology aimed to provide a comprehensive overview of blunt renal trauma cases, facilitating insights into effective management strategies and patient outcomes within the institution.

## 2. Materials and Methods

This retrospective study was conducted at the Emergency Clinical County Hospital in Brasov, Romania. Data were collected from patients admitted with high-grade blunt renal trauma (grade III or higher) between 1 January 2019, and 31 December 2025. Patients were eligible for inclusion if they were diagnosed with renal trauma resulting from blunt abdominal or thoracoabdominal injury, had a renal injury classified as grade III–V according to the American Association for the Surgery of Trauma (AAST) Organ Injury Scale, and were evaluated and managed at our institution during the study period. Patients were excluded if they had penetrating renal trauma, isolated renal trauma of AAST grade I–II, incomplete or unavailable medical records, or insufficient clinical or radiological data to establish the renal injury grade and subsequent management.

Recorded variables included the mechanism of injury, clinical and laboratory parameters, imaging findings, interventions performed, and patient outcomes. Renal injuries were classified using the American Association for the Surgery of Trauma (AAST) injury severity scale (Table 1).

Treatment selection was primarily based on the patient’s hemodynamic status, response to initial resuscitation, renal injury severity, associated injuries, and clinical and radiological findings. Hemodynamic status was assessed at the time of initial clinical evaluation using blood pressure, mean arterial pressure, heart rate, clinical signs of shock, and the patient’s response to initial resuscitative measures. Hemodynamic instability was defined in this study as a mean arterial pressure < 70 mmHg at initial clinical evaluation. Initial management included standard trauma resuscitation and supportive measures, as clinically indicated. Patients who remained hemodynamically stable or stabilized after initial resuscitation were considered for conservative management, consisting of the close monitoring of vital signs, serial clinical assessment, laboratory monitoring, and supportive treatment. Nephrectomy was performed in patients with persistent hemodynamic instability and/or severe renal injury requiring immediate operative control, taking into account associated injuries and the overall clinical condition.

Angioembolization was not available at our institution during the study period and, consequently, was not performed in any patient.

The study involved no risk to participants and was conducted in strict accordance with established ethical standards for human research. All procedures were reviewed and approved by the Ethics Commission of the Emergency County Hospital in Brasov, where the study was carried out (Ethics approval number: 12/06.02.2026). Informed consent was obtained from patients at the time of hospital admission, in accordance with the standard institutional admission procedures, which include consent for the use of clinical information for scientific research purposes. The research also adhered to the ethical guidelines of the College of Physicians in Romania and complied with the principles outlined in the Declaration of Helsinki (2013 revision).

Participant anonymity was rigorously safeguarded at all stages of the study.

Data collection Data were extracted from electronic medical records and included the following:

• Demographics: Age and gender;
• Mechanism of injury: Traffic accidents, falls or aggression;
• Injury Severity: Classification of renal injuries using the AAST grading system (grades III to V);
• Clinical Aspects: Symptoms and signs at presentation (hematuria, flank pain, and signs of shock), comorbidities;
• Imaging studies (CT–GE Optima 660);
• Laboratory tests: For each laboratory parameter, the value recorded during the admission was included in the analysis (hemoglobin levels, hematocrit levels, blood urea nitrogen, creatinine levels);
• Management Strategies: conservative or surgical approach;
• Outcomes: post-treatment evolution and mortality rates.

Statistical analysis was performed using MedCalc Statistical Software version 9.2.1.0 (MedCalc Software Ltd., Ostend, Belgium) and Microsoft Excel 2021 (Microsoft Corporation, Redmond, WA, USA). Continuous variables were assessed for distributional normality using the D’Agostino–Pearson test. Normally distributed continuous variables were compared between treatment groups using Welch’s independent-samples *t*-test, whereas non-normally distributed variables were compared using the Mann–Whitney U test. Categorical variables were compared using the chi-square test or Fisher’s exact test, as appropriate, particularly when expected cell frequencies were small. All tests were two-sided, and a *p*-value < 0.05 was considered statistically significant. Given the retrospective observational design and the substantial imbalance in injury severity between treatment groups, all between-group comparisons were considered unadjusted and descriptive, and no causal inference regarding treatment effects was made.

## 3. Results

**Figure 1:**
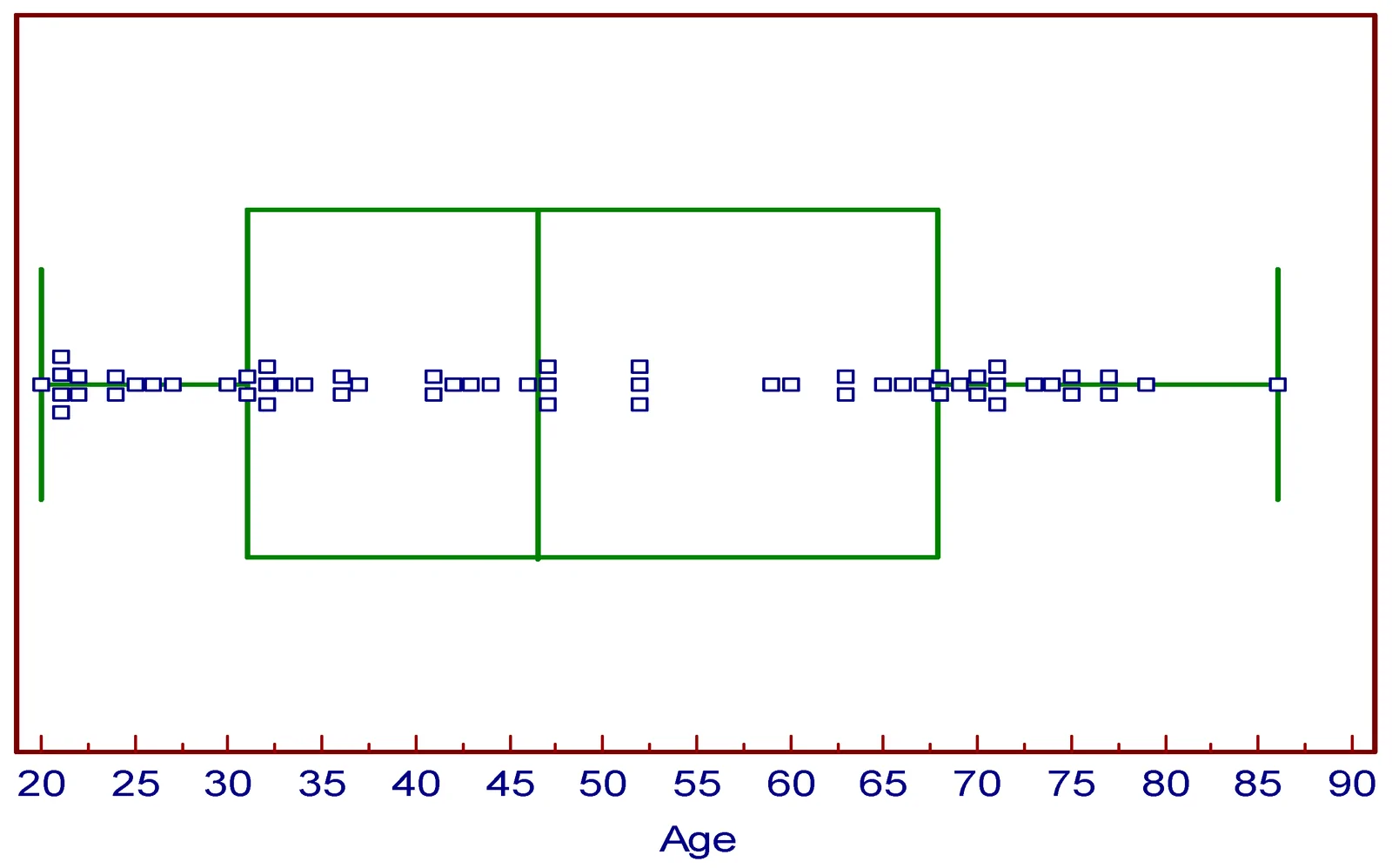
Boxplot showing the distribution by age.

A total of 58 patients were admitted with high-grade renal injury (grade III and above) during the study period, all cases of nonpenetrating renal trauma. Their ages varied from 20 to 86 years, with a mean age of 48.43 years. The highest incidence of renal trauma was observed in the 21–30 years age group (18.97%) and 31–40 years age group (18.97%). The standard error of the mean is 2.62, which reflects how precisely the mean age is estimated. (Figure 1) The skewness coefficient is 0.11 with a *p*-value of 0.69, indicating that the distribution is approximately symmetric (not significantly skewed). The kurtosis coefficient is −1.42 with a *p*-value of 0.06, suggesting that the distribution may be slightly flatter than normal, but not significantly so (Table 2).

Most of the patients with blunt renal trauma were males 81.03% and 18.97% females. Falls were the most common mechanism of injury, accounting for 50% of cases, followed by traffic accidents (25.86%) and assaults (24.14%).

The annual distribution of renal trauma cases showed considerable variability, with the highest proportion recorded in 2024 (24.14%), followed by 2025 (17.24%) and 2022 (15.52%), while the lowest proportions were observed in 2021 and 2023 (8.62% each).

**Figure 2:**
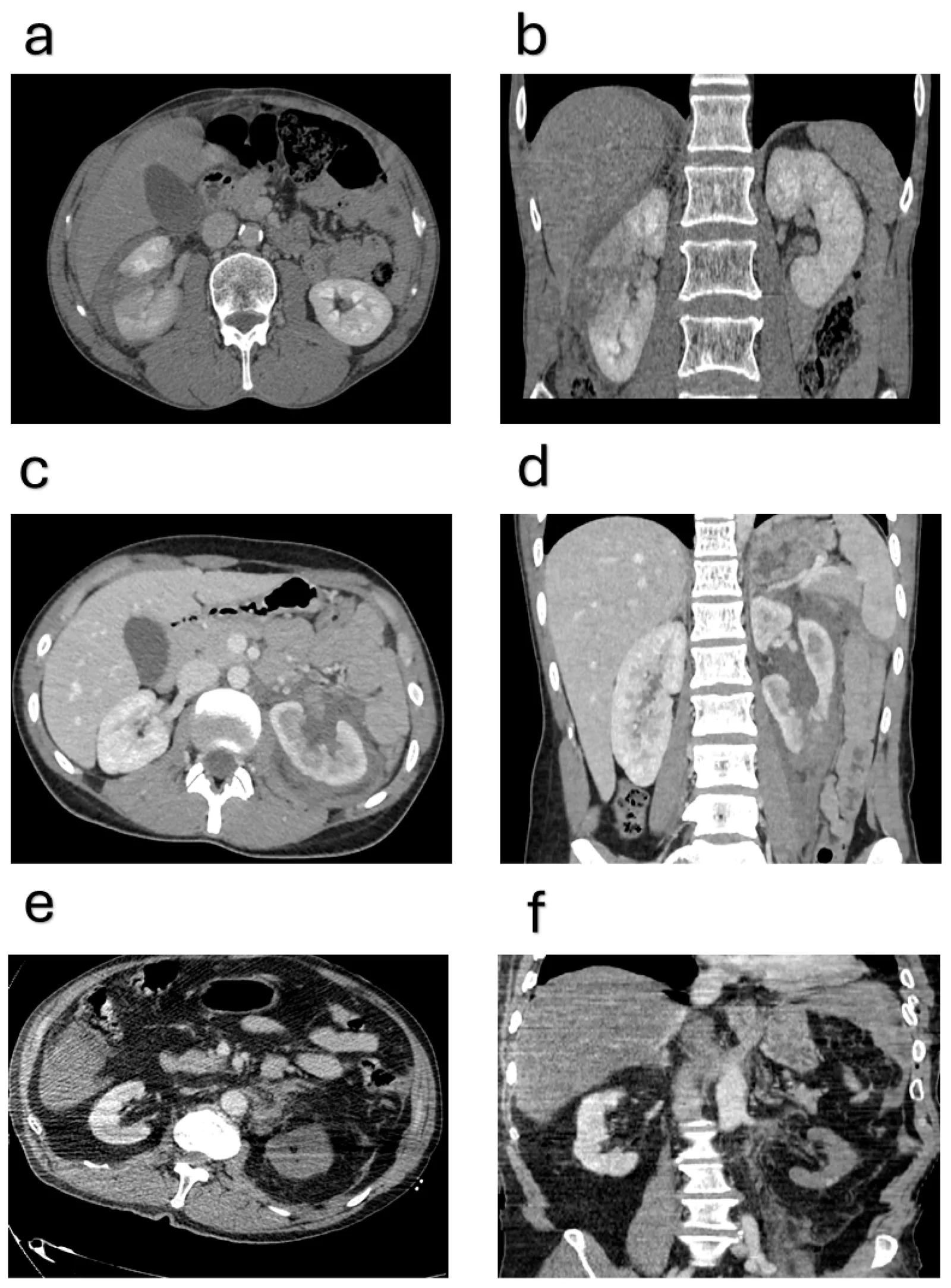
Contrast-enhanced CT scan showing: (**a**) right-kidney grade III injury—laceration and hematoma. Axial section. (**b**) Right-kidney grade III injury—laceration and hematoma. Coronal section. (**c**) Left-kidney grade IV injury—Parenchymal laceration extending through renal cortex, medulla and collecting system. Axial section. (**d**) Left-kidney grade IV injury—Parenchymal laceration extending through renal cortex, medulla and collecting system. Coronal section. (**e**) Left-kidney grade V injury—Completely shattered kidney. Avulsion of renal hilum (devascularized kidney). Axial section. (**f**) Left-kidney grade V injury—Completely shattered kidney. Avulsion of renal hilum (devascularized kidney). Coronal section.

The distribution of renal injuries according to the AAST classification was as follows: grade III (60.34%) (Figure 2a,b), grade IV (18.97%) (Figure 2c,d), and grade V (20.69%) (Figure 2e,f). All patients underwent contrast-enhanced CT scanning, with delayed excretory phase imaging, performed when clinically indicated.

Referring to the right/left side, the most affected kidney was the left kidney—32 patients, (55.17%). The right kidney was affected in 26 patients (44.83%).

At presentation, all patients had flank pain, 38 of them presented hematuria (macroscopic or microscopic hematuria). The MAP values range from 31.33 mmHg to 118.33 mmHg. The average (mean) MAP is 88.91. The median MAP is 93.16 mmHg, meaning that half of the individuals have MAP values below 93.16 mmHg. Hemodynamic shock, defined as a MAP below 70 mmHg, was documented at the time of initial clinical evaluation in 15.51% of patients in the cohort.

**Figure 3:**
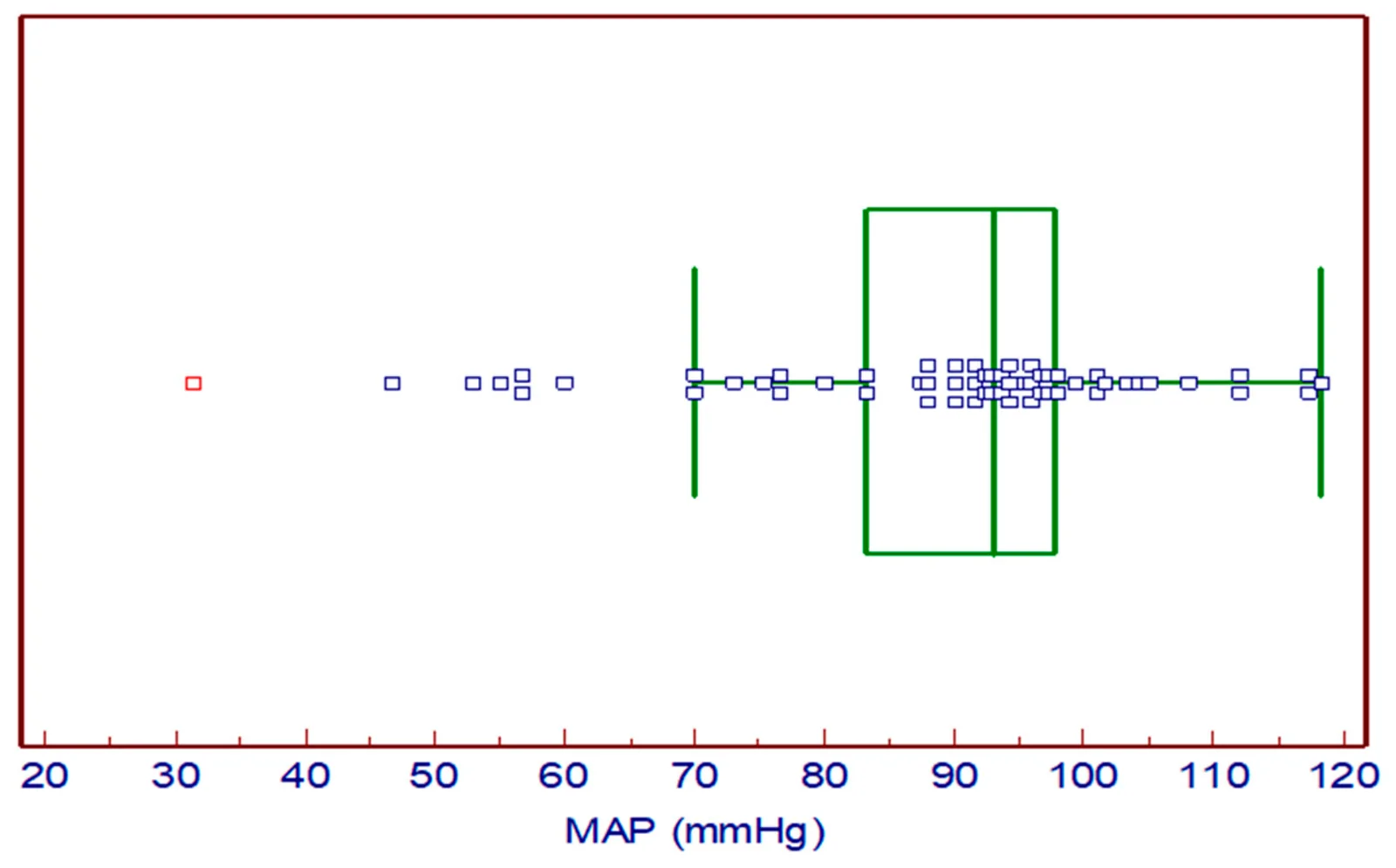
Boxplot showing the distribution of MAP (mmHg).

The negative skewness coefficient (−1.09, *p* = 0.0016), indicates a left-skewed distribution, meaning that several low MAP values pulled the mean downward. The coefficient of kurtosis was 1.37 (*p* = 0.0664), suggesting a tendency toward a more peaked distribution with heavier tails than a normal distribution, although this result was not statistically significant (Figure 3, Table 3).

**Figure 4:**
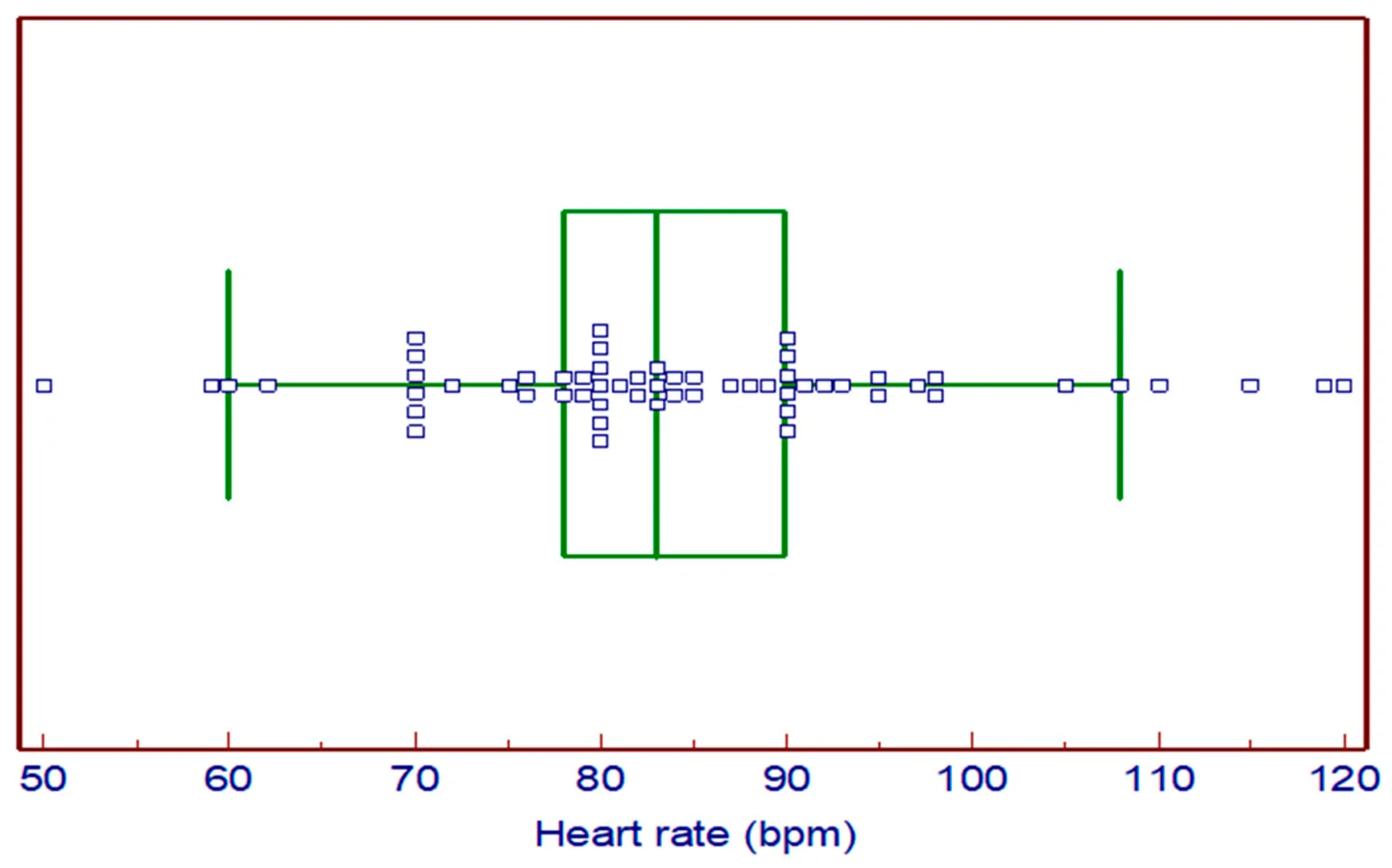
Boxplot showing the distribution of heart rate (bpm).

The heart rate values range from 50 bpm to 120 bpm. The average (mean) heart rate is 84.41. The median heart rate is 83 bpm. The skewness coefficient is 0.39 with a *p*-value of 0.19, indicating the distribution is slightly right-skewed, but not significantly. The kurtosis coefficient is 0.82 with a *p*-value of 0.18, suggesting no significant difference in peakedness compared to a normal distribution (Figure 4, Table 4).

**Figure 5:**
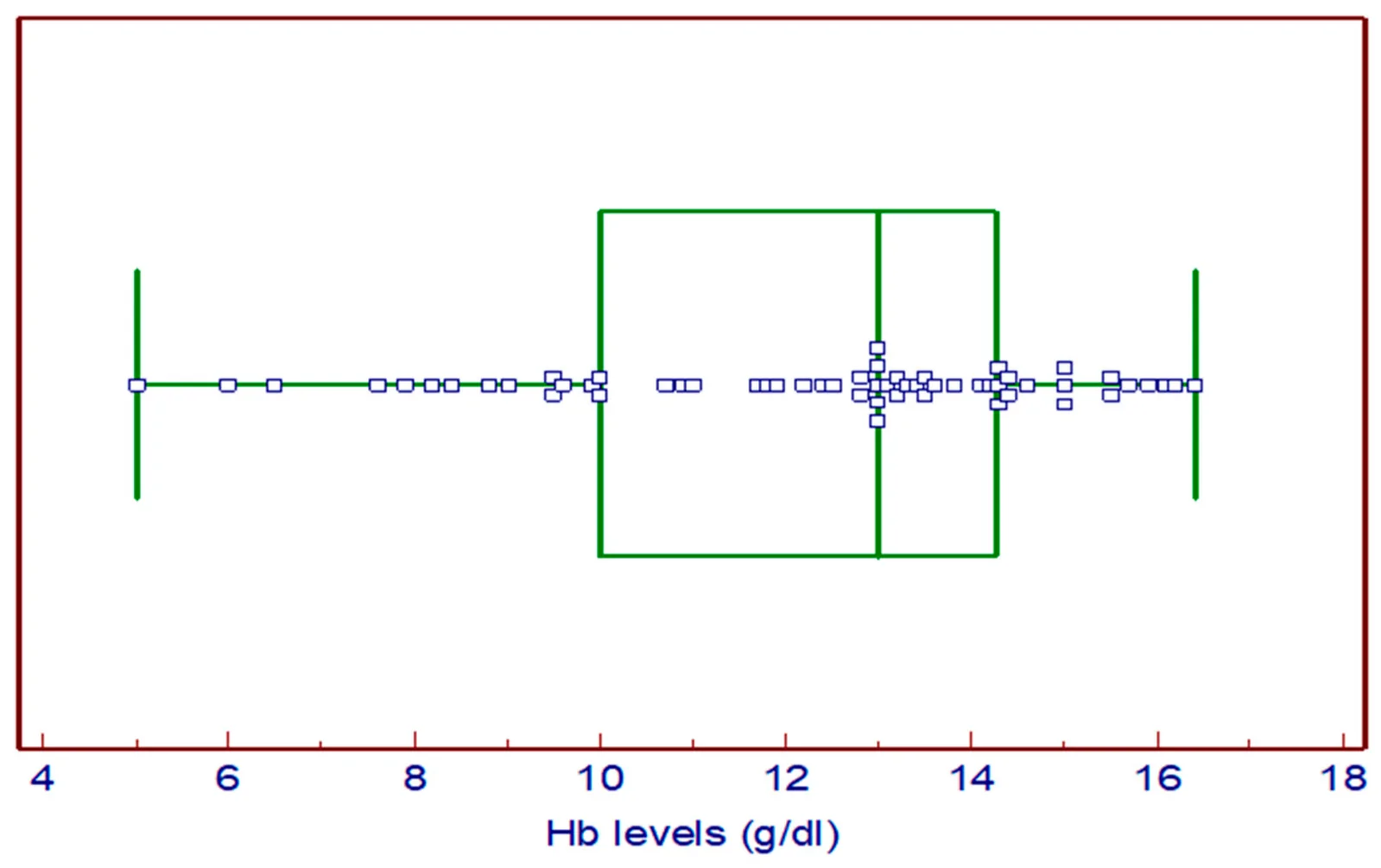
Boxplot showing the distribution of hemoglobin level (g/dL).

HB values ranged from 5.0 g/dL to 16.4 g/dL, indicating a wide variation in hemoglobin concentration among participants. The median HB value was 13.0 g/dL (95% CI: 11.96– 13.50 g/dL), slightly higher than the mean. This difference is consistent with the negative skewness coefficient (−0.75, *p* = 0.0204), which indicates a moderately left-skewed distribution, meaning that lower HB values influenced the mean. The coefficient of kurtosis (0.08, *p* = 0.7244) was close to zero and not statistically significant, indicating that the distribution has a shape similar to a normal distribution (Figure 5, Table 5).

**Figure 6:**
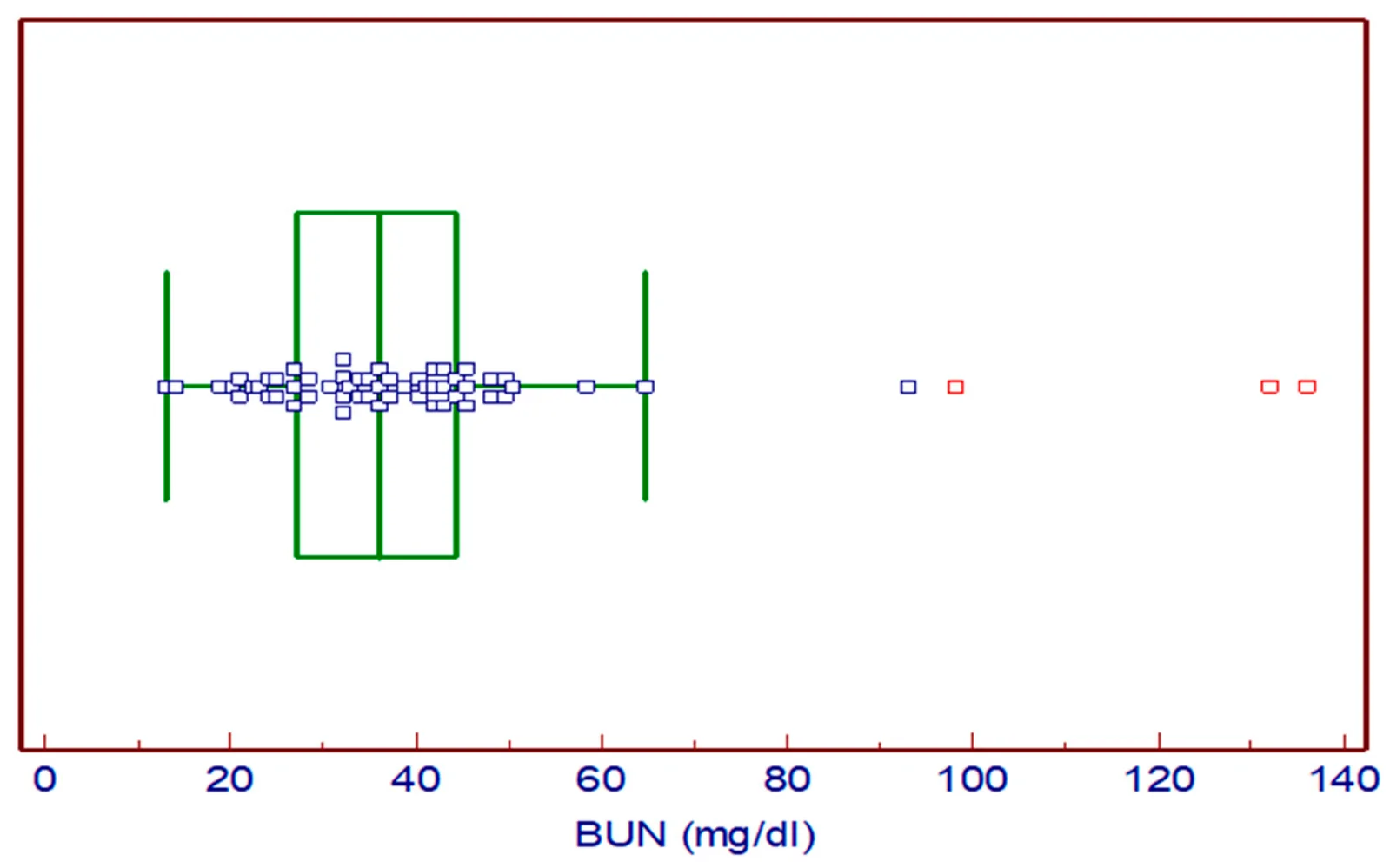
Boxplot showing the distribution of BUN (mg/dL).

BUN values ranged from 13.0 mg/dL to 136.1 mg/dL, demonstrating a very wide distribution of results within the study population. The median BUN value was 36.0 mg/dL (95% CI: 32.32–42.00 mg/dL), which is lower than the mean. This difference reflects the presence of high BUN values that increased the average. The coefficient of skewness (2.61, *p* < 0.0001) indicates a strong positive skew, meaning that the distribution is heavily shifted toward higher values. The coefficient of kurtosis (7.99, *p* < 0.0001) indicates a markedly leptokurtic distribution, characterized by a sharp peak and heavy tails, suggesting the presence of extreme values or outliers (Figure 6, Table 6).

**Figure 7:**
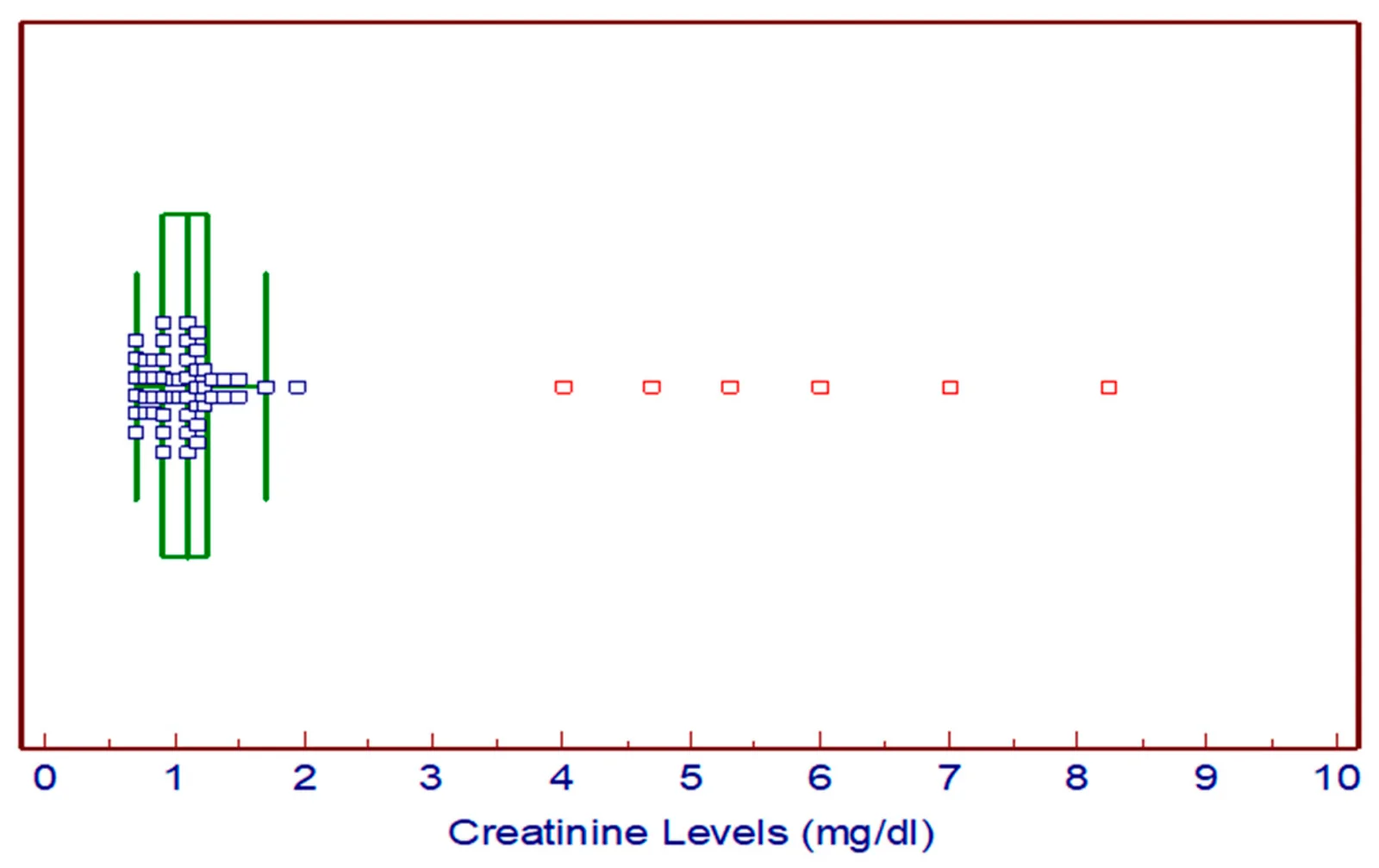
Boxplot showing the distribution of serum creatinine levels (mg/dL).

Serum creatinine values ranged from 0.70 mg/dL to 8.23 mg/dL, indicating a very broad range of renal function within the study population. The median creatinine level was 1.10 mg/dL (95% CI: 0.93–1.20 mg/dL), which is substantially lower than the mean. This difference reflects the influence of several markedly elevated creatinine values on the average. The coefficient of skewness (2.98, *p* < 0.0001) indicates a strong positive skew, showing that the distribution is heavily shifted toward higher values. The coefficient of kurtosis (8.39, *p* < 0.0001) indicates a strongly leptokurtic distribution, characterized by heavy tails and the likely presence of outliers or extreme high values (Figure 7, Table 7).

The majority of the patients (41) had associated organ injuries (70.69%). The most associated injuries were ribs fractures (26 patients), followed by spleen injuries (17 patients), liver injuries (15 patients), and brain injuries (8 patients).

Most patients did not have comorbidities; however, pre-existing anticoagulant therapy was reported in 3/35 (8.6%) grade III, 2/11 (18.2%) grade IV, and 7/12 (58.3%) grade V injuries, accounting for 12/58 (20.69%) patients overall; although anticoagulation may contribute to increased bleeding, larger perirenal hematomas, and hemodynamic instability following renal injury, this association cannot be established from the present data.

Additionally, 5.17% of patients were undergoing chronic dialysis treatment due to chronic kidney disease, indicating the presence of pre-existing severe renal dysfunction in a small proportion of cases. These patients may represent a particularly vulnerable sub-group, with limited renal reserve and potentially more complex post-traumatic management.

Furthermore, 20.69% of patients had renal cysts, which may alter renal parenchymal architecture and could influence the susceptibility to traumatic injury.

The management of renal trauma was conservative in 40 patients (68.97%) and nephrectomy was performed in 18 cases (31.03%).

Non-operative management was used in 32 patients with grade III renal injuries. Nephrectomy was performed in only three cases, all of which involved patients who were hemodynamically unstable and had associated high-grade splenic injuries. Regarding patients with grade IV renal trauma, non-operative management was performed in eight patients (72.73%). All patients with grade V renal trauma underwent emergency nephrectomy. Patients were classified according to the treatment ultimately received. No patient initially selected for conservative management subsequently required nephrectomy.

**Figure 8:**
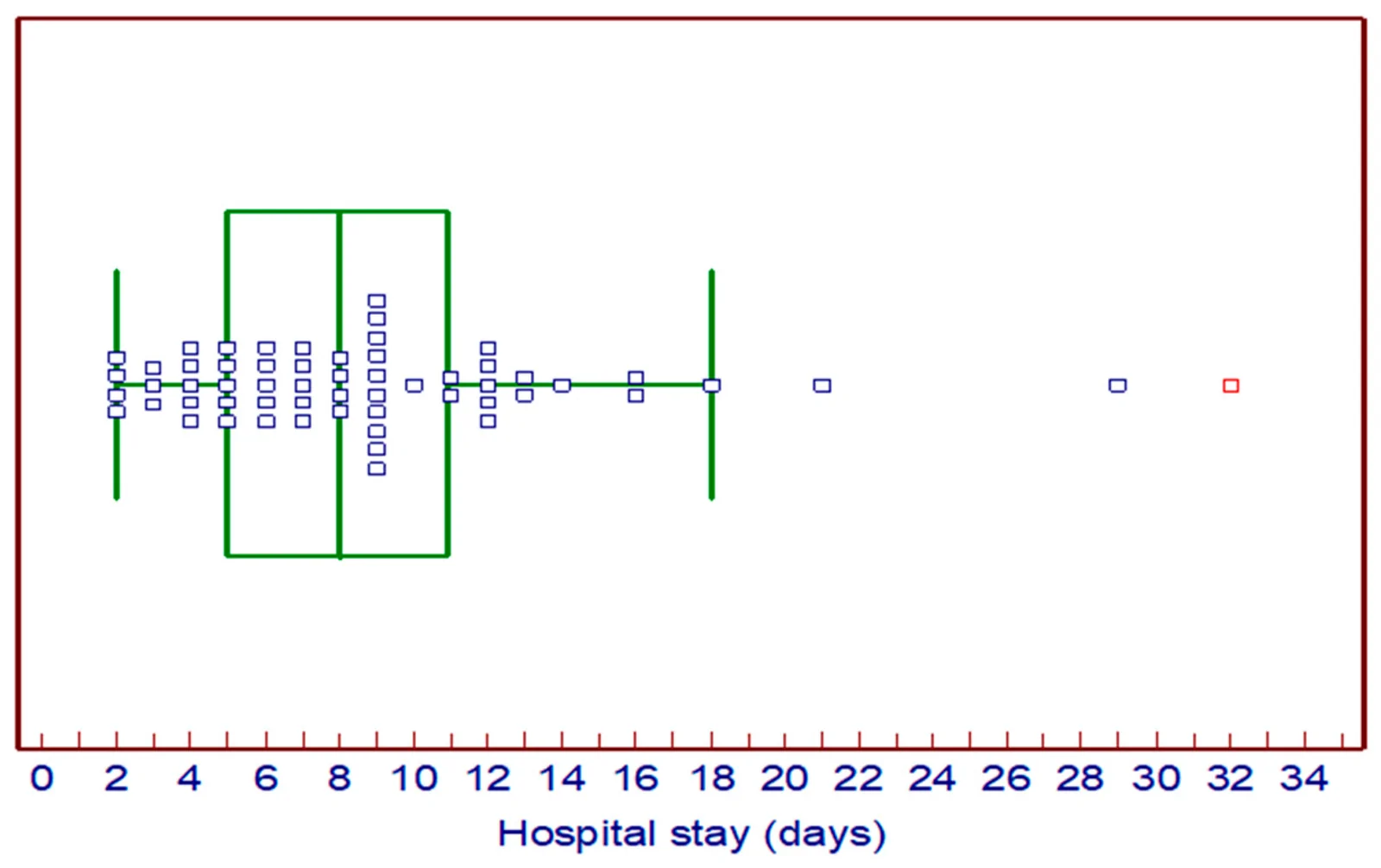
Boxplot showing the distribution of hospital stay (days).

The mean hospital length of stay was 8.84 days, ranging from 2 to 32 days (Figure 8). The positive skewness indicates a long right tail, reflecting that some patients had substantially longer stays. The high kurtosis suggests heavy tails, with more extreme values than expected in a normal distribution. The standard error of the mean (SEM) was 0.76. (Table 8).

Among the 58 patients included in the analysis, 18 (31.03%) underwent nephrectomy, whereas 40 (68.97%) were managed conservatively. The demographic, clinical, and laboratory characteristics of the two groups are presented in Table 9.

Values are presented as mean ± standard deviation, median (interquartile range), or *n* (%), as appropriate. *p*-values represent unadjusted between-group comparisons and should be interpreted descriptively because of the substantial imbalance in AAST injury severity between treatment groups.

Hemoglobin levels were lower in the nephrectomy group than in the conservative management group (9.76 ± 2.28 vs. 13.44 ± 2.11 g/dL; *p* < 0.001). Serum creatinine was higher in the nephrectomy group (median 1.28 [IQR 1.13–1.89] vs. 0.995 [IQR 0.845–1.178] mg/dL; *p* = 0.00077).

Hemodynamic parameters showed a trend toward lower blood pressure in the nephrectomy group, with median values of 88.17 (IQR 57.5–98.08) versus 93.83 (IQR 89.58–97.5) mmHg in the conservative management group; however, the difference was not statistically significant (*p* = 0.116).

A significant difference was observed in the distribution of AAST injury grades between the two treatment groups (*p* < 0.001). Conservative management was used in 32/35 (91.4%) grade III and 8/11 (72.7%) grade IV injuries, whereas all grade V injuries (12/12; 100%) underwent nephrectomy.

Mortality occurred in four patients (22.2%) in the nephrectomy group and in one patient (2.5%) in the conservative management group.

Patients in the nephrectomy group had a higher median age than those in the conservative management group (59.5 [IQR 32.25–72.5] vs. 43.5 [IQR 29.25–63.5] years), although the difference was not statistically significant (*p* = 0.110).

The nephrectomy group had substantially more severe renal injuries, lower hemoglobin levels, higher serum creatinine values, and higher mortality. These unadjusted differences should be interpreted descriptively, given the marked imbalance in baseline injury severity between the treatment groups.

The overall mortality rate was 8.62%. (grade III: 5.71%, grade IV: 0%, grade V: 25.00%). All were polytrauma patients, and three of the five had severe renal injuries (AAST grade V). The patients were predominantly elderly (median age, 70 years), and marked physiological and laboratory abnormalities were observed in several cases.

Two patients had profound anemia (hemoglobin 5.0 and 6.0 g/dL) associated with severe renal dysfunction (creatinine 5.30 and 6.00 mg/dL). One patient presented with extreme hemodynamic instability, with a blood pressure of 50/22 mmHg and a mean arterial pressure of 31.3 mmHg. Three patients with AAST grade V injuries underwent nephrectomy, while another patient with grade III injury underwent nephrectomy and splenectomy. One patient with a grade III renal injury was managed conservatively and subsequently died due to a severe associated head injury.

Associated injuries were frequent, particularly thoracic, cranial, hepatic, and splenic trauma. The two treatment groups differed substantially in baseline injury severity, with all 12 patients with AAST grade V injuries belonging to the nephrectomy group, whereas no grade V patient was managed conservatively.

## 4. Discussion

High-grade renal trauma, defined by AAST as grades III–V, represents a spectrum of injuries with varying clinical implications and management challenges. Traditionally, these injuries were considered indications for surgical treatment due to concerns regarding hemorrhage, urinary extravasation, and loss of renal function. However, growing evidence over the past two decades has demonstrated that a significant proportion of high-grade renal injuries following blunt trauma can be managed successfully with nonsurgical strategies in hemodynamically stable patients [5,6,11].

Grade III renal injuries have consistently shown high success rates with conservative management. Observation with close clinical and radiological monitoring is now widely accepted as the standard of care for these patients. Surgical intervention is rarely required, unless trauma is associated with ongoing bleeding and hemodynamic instability [10,12].

Management of grade IV renal injuries remains more controversial due to the presence of collecting system involvement or segmental vascular injury. Several contemporary studies have reported favorable outcomes with non-operative management in stable patients, particularly with the availability of angioembolization and ureteric stenting.

These techniques allow effective control of hemorrhage and urinary extravasation while preserving renal parenchyma, thereby reducing the need for nephrectomy [6,13,14].

Grade V renal injuries, which include shattered kidneys and main renal vascular injuries, represent the most severe grade and are associated with higher rates of surgical treatment and complications. Nevertheless, selective nonoperative management has been reported in carefully selected patients who remain hemodynamically stable. Advances in imaging, intensive care monitoring, and interventional radiology have contributed to improved renal salvage rates, challenging the traditional paradigm of mandatory surgical exploration [15,16].

Hemodynamic stability remains the most important factor of management strategy across all high-grade renal injuries. Patients with persistent instability despite resuscitation require prompt surgical intervention. Injury grade alone should not dictate the management strategy, as associated injuries, contrast extravasation on imaging, transfusion requirements, and clinical trajectory play equally important roles in decision-making. In our cohort of 58 patients, non-operative management was used in 68.97% of cases. In our cohort, this approach was used predominantly in patients with grade III injuries, among whom 32 of 35 patients (91.43%) were managed conservatively.

Similarly, 8 of 11 patients (72.73%) with grade IV injuries were managed without surgery. In contrast, all patients with grade V injuries underwent emergency nephrectomy, highlighting the substantially different clinical profile of the most severe renal injuries.

These findings are consistent with the contemporary shift toward the selective non-operative management of renal trauma. Dagenais et al., in a population-based analysis of more than 21,000 patients, reported an increase in non-operative management from 65.5% to 81.8% over an 11-year period, although their cohort included renal injuries of all grades and both blunt and penetrating mechanisms. Similarly, a nationwide Korean study reported that 85.8% of high-grade renal injuries were managed conservatively, supporting the concept that injury grade alone should not constitute an indication for operative exploration. Our findings extend these observations to a cohort composed exclusively of high-grade blunt renal trauma and are particularly relevant to settings where angioembolization is unavailable. In our cohort, a substantial proportion of grade III–IV injuries were managed without nephrectomy.

Importantly, all patients with grade V injuries in our cohort underwent emergency nephrectomy, reflecting the severity of these injuries and the clinical circumstances at presentation [17,18].

The findings of the present study support an individualized, selective approach to high-grade renal trauma, emphasizing renal preservation whenever it is appropriate. Non-operative management may be appropriate in carefully selected patients, particularly those who remain hemodynamically stable or stabilize after initial resuscitation [7,8,19].

Angioembolization plays a crucial role in the contemporary management of high-grade renal trauma, particularly in hemodynamically stable patients with evidence of active bleeding or segmental vascular injury. Multiple studies have demonstrated that this minimally invasive technique allows effective hemorrhage control, reduces the need for surgical exploration, and significantly improves renal preservation rates. As a result, angioembolization has become an integral component of non-operative management algorithms for high-grade blunt renal injuries in well-equipped trauma centers. In the absence of angioembolization, treatment decisions rely heavily on meticulous clinical monitoring, intensive supportive care, and selective surgical intervention in cases of hemodynamic instability or clinical deterioration [13,20].

These findings highlight the need for broader availability of interventional radiology services in trauma centers managing complex renal injuries. The incorporation of angioembolization into institutional trauma protocols could further optimize outcomes, reduce surgical morbidity, and align management strategies with current evidence-based recommendations [21,22]. High-grade blunt renal trauma no longer mandates immediate surgical exploration in all cases, as modern trauma management has demonstrated that a selective non-operative approach can be used in appropriately selected patients.

Careful assessment of hemodynamic stability, extent of renal injury, associated organ damage, and continuous clinical monitoring allows many patients to recover without the need for nephrectomy or invasive surgery. The increasing use of advanced imaging techniques, interventional radiology, and intensive supportive care has further supported the use of conservative management in appropriately selected patients by controlling complications while preserving renal function [17,23].

Among patients with grade V renal trauma, 58.33% were receiving anticoagulant therapy prior to the traumatic event. Although this finding is notable, its clinical significance remains uncertain, as no statistical analysis was performed to determine whether preexisting anticoagulation was associated with renal injury severity or clinical outcomes.

Anticoagulant therapy may theoretically contribute to increased bleeding following trauma; however, this hypothesis cannot be confirmed based on the present data. Therefore, this observation should be considered exploratory and interpreted with caution.

Further studies incorporating appropriate control groups and multivariable analyses are needed to determine whether pre-existing anticoagulation independently influences the severity or outcomes of high-grade renal trauma [6,24].

This study has important limitations that should be acknowledged. First, its retrospective design is inherently associated with the potential for selection and information bias, as the analysis was based on data available in the medical records. Second, the study was conducted at a single tertiary referral center, which may limit the generalizability of the findings to other institutions with different patient populations, trauma systems, and management protocols. Third, long-term follow-up was not systematically available for all patients; therefore, delayed complications and long-term renal outcomes could not be comprehensively assessed. Finally, angioembolization was not available at our institution during the study period and was therefore not performed, precluding an assessment of its potential role in the management of high-grade renal trauma within our cohort. Despite these limitations, the study provides data on the outcomes of high-grade blunt renal trauma managed in a setting in which non-operative management was the predominant treatment strategy.

## 5. Conclusions

In this retrospective cohort, conservative management was frequently used among patients with AAST grade III–IV renal injuries who were hemodynamically stable or stabilized after initial resuscitation. Treatment selection was based on hemodynamic status, injury severity, associated injuries, and clinical evolution. Because of the substantial baseline imbalance in injury severity between the treatment groups, this study does not allow causal conclusions regarding the relative effects of conservative management and nephrectomy on clinical outcomes. The findings support an individualized approach based on hemodynamic status, injury severity, associated injuries, and clinical evolution rather than a comparison of treatment effectiveness.

Overall, multidisciplinary, individualized patient management is the key to achieving optimal clinical outcomes in high-grade blunt renal trauma.

Future studies should focus on prospective, multicenter evaluations to refine selection criteria for nonoperative management, particularly in grade IV and V injuries. Longterm follow-up assessing renal function, hypertension, and quality of life is also essential to better define the true impact of conservative versus interventional treatments in high grade renal trauma.

## Figures and Tables

**Table 1:** AAST classification (2025).

Classification	Description
Grade I	– Subcapsular hematoma < 3.5 cm without active bleeding – Parenchymal contusion without laceration
Grade II	– Parenchymal laceration length < 2.5 cm – Hematoma Rim Distance (HRD) < 3.5 cm without active bleeding
Grade III	– Parenchymal laceration length ≥ 2.5 cm – HRD ≥ 3.5 cm without active bleeding – Partial kidney infarction – Vascular injuries without active bleeding – Laceration extending into urinary collecting system and/or urinary extravasation
Grade IV	– Active bleeding from kidney – Pararenal extension of hematoma – Complete/near-complete kidney infarction without active bleeding – Multifragmented kidney without active bleeding – Complete/near-complete ureteropelvic junction disruption
Grade V	– Main renal artery or vein laceration or transection with active bleeding – Complete/near-complete kidney infarction with active bleeding – Multifragmented kidney with active bleeding

**Table 2:** Distribution of patients by age (years).

Variable	Age
Sample size	58
Lowest value	20.0000
Highest value	86.0000
Arithmetic mean	48.4310
95% CI for the mean	43.1829 to 53.6792
Median	46.5000
95% CI for the median	36.0000 to 62.3520
Variance	398.3899
Standard deviation	19.9597
Relative standard deviation	0.4121 (41.21%)
Standard error of the mean	2.6208
Coefficient of skewness	0.1183 (*p* = 0.6936)
Coefficient of kurtosis	−1.4274 (*p* = 0.0602)
D’Agostino-Pearson test for normal distribution	Normality was not rejected (*p* = 0.1582)

**Table 3:** Distribution of patients by mean arterial pressure (MAP) (mmHg).

Variable	MAP (mmHg)
Sample size	58
Lowest value	31.3300
Highest value	118.3300
Arithmetic mean	88.9193
95% CI for the mean	84.2316 to 93.6070
Median	93.1650
95% CI for the median	90.0000 to 96.2587
Variance	317.8492
Standard deviation	17.8283
Relative standard deviation	0.2005 (20.05%)
Standard error of the mean	2.3410
Coefficient of skewness	−1.0915 (*p* = 0.0016)
Coefficient of kurtosis	1.3736 (*p* = 0.0664)
D’Agostino-Pearson test for normal distribution	reject Normality (*p* = 0.0013)

**Table 4:** Distribution of patients by heart rate (bpm).

Variable	Heart Rate (bpm)
Sample size	58
Lowest value	50.0000
Highest value	120.0000
Arithmetic mean	84.4138
95% CI for the mean	80.7522 to 88.0754
Median	83.0000
95% CI for the median	80.0000 to 88.7840
Variance	193.9310
Standard deviation	13.9259
Relative standard deviation	0.1650 (16.50%)
Standard error of the mean	1.8286
Coefficient of skewness	0.3982 (*p* = 0.1951)
Coefficient of kurtosis	0.8255 (*p* = 0.1851)
D’Agostino-Pearson test for normal distribution	Normality was not rejected (*p* = 0.1795

**Table 5:** Distribution of patients by Hb levels (g/dL).

Variable	HB Levels (g/dL)
Sample size	58
Lowest value	5.0000
Highest value	16.4000
Arithmetic mean	12.2948
95% CI for the mean	11.5726 to 13.0171
Median	13.0000
95% CI for the median	11.9648 to 13.5000
Variance	7.5458
Standard deviation	2.7470
Relative standard deviation	0.2234 (22.34%)
Standard error of the mean	0.3607
Coefficient of skewness	−0.7525 (*p* = 0.0204)
Coefficient of kurtosis	−0.0829 (*p* = 0.7244)
D’Agostino-Pearson test for normal distribution	Normality was not rejected (*p* = 0.0639)

**Table 6:** Distribution of patients by blood urea nitrogen levels (mg/dL).

Variable	BUN (mg/dL)
Sample size	58
Lowest value	13.0000
Highest value	136.1000
Arithmetic mean	40.9347
95% CI for the mean	34.7802 to 47.0891
Median	36.0000
95% CI for the median	32.3216 to 42.0000
Variance	547.8644
Standard deviation	23.4065
Relative standard deviation	0.5718 (57.18%)
Standard error of the mean	3.0734
Coefficient of skewness	2.6099 (*p* < 0.0001)
Coefficient of kurtosis	7.9903 (*p* < 0.0001)
D’Agostino-Pearson test for normal distribution	reject Normality (*p* < 0.0001)

**Table 7:** Distribution of patients by serum creatinine levels (mg/dL).

Variable	CREATININE Levels (mg/dL)
Sample size	58
Lowest value	0.7000
Highest value	8.2300
Arithmetic mean	1.5626
95% CI for the mean	1.1506 to 1.9746
Median	1.1000
95% CI for the median	0.9343 to 1.2000
Variance	2.4554
Standard deviation	1.5670
Relative standard deviation	1.0028 (100.28%)
Standard error of the mean	0.2058
Coefficient of skewness	2.9830 (*p* < 0.0001)
Coefficient of kurtosis	8.3908 (*p* < 0.0001)
D’Agostino-Pearson test for normal distribution	reject Normality (*p* < 0.0001)

**Table 8:** Distribution of patients by hospital length of stay (days).

Variable	Hospital Stay (Days)
Sample size	58
Lowest value	2.0000
Highest value	32.0000
Arithmetic mean	8.8448
95% CI for the mean	7.3110 to 10.3786
Median	8.0000
95% CI for the median	6.0000 to 9.0000
Variance	34.0281
Standard deviation	5.8334
Relative standard deviation	0.6595 (65.95%)
Standard error of the mean	0.7660
Coefficient of skewness	1.9657 (*p* < 0.0001)
Coefficient of kurtosis	5.3355 (*p* = 0.0002)
D’Agostino-Pearson test for normal distribution	reject Normality (*p* < 0.0001)

**Table 9:** Descriptive comparison of demographic, clinical, laboratory, and outcome characteristics according to treatment received.

Variable	Nephrectomy (*n* = 18)	Conservative Management (*n* = 40)	*p*-Value
Age, years	59.5 (32.25–72.5)	43.5 (29.25–63.5)	0.110
Mean arterial pressure, mmHg	88.17 (57.5–98.08)	93.83 (89.58–97.5)	0.116
Heart rate, bpm	88.56 ± 19.25	82.55 ± 10.51	0.227
Hemoglobin, g/dL	9.76 ± 2.28	13.44 ± 2.11	<0.001
Creatinine, mg/dL	1.28 (1.13–1.89)	0.995 (0.845–1.178)	0.00077
Hematuria, *n* (%)	9 (50%)	29 (72.5%)	0.136
AAST grade			<0.001
III	3 (16.7%)	32 (80%)	
IV	3 (16.7%)	8 (20%)	
V	12 (66.6%)	0 (0%)	
Mortality, *n* (%)	4 (22.2%)	1 (2.5%)	0.029
Length of hospital stay, days	9 (7–12)	7 (5–9)	0.120

## Data Availability

The original contributions presented in this study are included in the article. Further inquiries can be directed to the corresponding author.
